# Dataset of MIGRAME Project (Global Change, Altitudinal Range Shift and Colonization of Degraded Habitats in Mediterranean Mountains)

**DOI:** 10.3897/phytokeys.56.5482

**Published:** 2015-10-01

**Authors:** Antonio Jesús Pérez-Luque, Regino Zamora, Francisco Javier Bonet, Ramón Pérez-Pérez

**Affiliations:** 1 Laboratorio de Ecología (iEcolab), Instituto Interuniversitario de Investigación del Sistema Tierra en Andalucía (CEAMA), Universidad de Granada, Avenida del Mediterráneo s/n, 18006, Granada, Spain; 2 Grupo de Ecología Terrestre, Departamento de Ecología, Universidad de Granada, Facultad de Ciencias, Campus de Fuentenueva s/n, 18071, Granada, Spain

**Keywords:** *Quercus pyrenaica* forests, altitudinal migration, colonization of abandoned croplands, global change, Sierra Nevada (Spain), occurrence data, measurement data

## Abstract

In this data paper, we describe the dataset of the Global Change, Altitudinal Range Shift and Colonization of Degraded Habitats in Mediterranean Mountains (MIGRAME) project, which aims to assess the capacity of altitudinal migration and colonization of marginal habitats by *Quercus pyrenaica* Willd. forests in Sierra Nevada (southern Spain) considering two global-change drivers: temperature increase and land-use changes. The dataset includes information of the forest structure (diameter size, tree height, and abundance) of the *Quercus pyrenaica* ecosystem in Sierra Nevada obtained from 199 transects sampled at the treeline ecotone, mature forest, and marginal habitats (abandoned cropland and pine plantations). A total of 3839 occurrence records were collected and 5751 measurements recorded. The dataset is included in the Sierra Nevada Global-Change Observatory (OBSNEV), a long-term research project designed to compile socio-ecological information on the major ecosystem types in order to identify the impacts of global change in this mountain range.

## Project details

### Project title

Global Change, altitudinal range shift and colonization of degraded habitats in Mediterranean mountains (MIGRAME)

### Personnel

Regino Jesús Zamora Rodríguez (Principal Investigator, University of Granada)

### Funding

The project MIGRAME (RNM-6734) was funded by the Excellence Research Group Programme of the Andalusian Government (Spain).

### Rationale

Currently, there is strong scientific evidence of the effects of global change on natural systems (Parmesan 2006, Rosenzweig et al. 2008, García et al. 2014, O’Connor et al. 2015). Some ecological processes are being altered due to the changing climate, such as species distribution (Thuiller et al. 2005, Lenoir et al. 2008), phenology (Parmesan and Yohe 2003, Gordo and Sanz 2010, Wolkovich et al. 2014), ecological interactions (Hughes 2000, Suttle et al. 2007); among others. Land-use changes and climate change are the most important drivers of biodiversity shifts (Sala et al. 2000).

One of the most obvious biotic responses from global warming are the latitudinal and altitudinal shifts of species and communities (Allen and Breshears 1998, Jump and Peñuelas 2005, Lenoir et al. 2008). Species tend to expand into new areas that are becoming favourable, and retract from those that turn hostile. In consideration of two main drivers of global change (climatic warming and land abandonment), an understanding of the dynamics of altitudinal migration and colonization of marginal habitats is critical in order to develop effective forest-management strategies.

The project Global Change, altitudinal range shift, and colonization of degraded habitats in Mediterranean mountains (MIGRAME) was designed to assess the capacity of altitudinal migration and colonization of marginal habitats by a Mediterranean forest ecosystem (Zamora et al. 2013, Benito et al. 2013). This assessment considers two global change drivers: temperature increase and land-use changes. In so doing, this project analyzes the pattern of altitudinal migration and colonization of marginal habitats by a vulnerable ecosystem in a Mediterranean mountain region, which represents the rear edge of their distribution: forests of *Quercus pyrenaica* Willd.

The Mediterranean region has shown broad climate shifts in the past (Luterbacher et al. 2006) and is potentially vulnerable to forthcoming climatic changes (Pacifici et al. 2015), being considered a key region in future climate-change projections (Giorgi 2006, Giorgi and Lionello 2008). Concomitantly, land-use changes are considered a major driver of vegetation change (McGill 2015). This is especially relevant in Mediterranean region, which has undergone intense antrophic activities for millennia (Padilla et al. 2010) shaping the current landscape (Valbuena-Carabaña et al. 2010).

In this context, Mediterranean ecosystems are considered natural laboratories in which to study global change, due to their high sensitivity to global-change drivers (Matesanz and Valladares 2014, Doblas-Miranda et al. 2015).

### Study area descriptions/descriptor

The target ecosystem of the project encompasses the Pyrenean oak forests (*Quercus pyrenaica* Willd.) of Sierra Nevada.

Sierra Nevada is a high-mountain range located in southern Spain (37°N, 3°W) with altitudes of between 860 m and 3482 m a.s.l. The climate is Mediterranean, characterized by cold winters and hot summers, with pronounced summer drought (July-August). The Sierra Nevada mountain range hosts a high number of endemic plant species (c. 80) (Lorite et al. 2007) for a total of 2,100 species of vascular plants (25% and 20% of Spanish and European flora, respectively), and thus it is considered one of the most important biodiversity hotspots in the Mediterranean region (Blanca et al. 1998). This mountain area has 27 habitat types (listed in the European Union Habitat Directive) harbouring 31 animal species (20 birds, 5 mammals, 4 invertebrates, 2 amphibians and reptiles) and 20 plant species listed in the Annex I and II of EU Habitat and Bird Directives. Sierra Nevada has several types of legal protection: Biosphere Reserve MAB Committee UNESCO; Special Protection Area and Site of Community Importance (Natura 2000 network); and National Park. There are 61 municipalities with more than 90,000 inhabitants. The main economic activities are agriculture, tourism, beekeeping, mining, and skiing (Bonet et al. 2010).

For a description of the Pyrenean oak forests in Sierra Nevada, see *Study extent description* section.

### Design description

The specific aims of the MIGRAME project are:

- To analyse the relevance of altitudinal migration at the leading edge (high elevation) of the range distribution of Pyrenean oak formation.
- To analyse the importance of the recolonization process of marginal habitats (abandoned croplands and pine plantations) close to Pyrenean oak formation.

**Figure 1.**
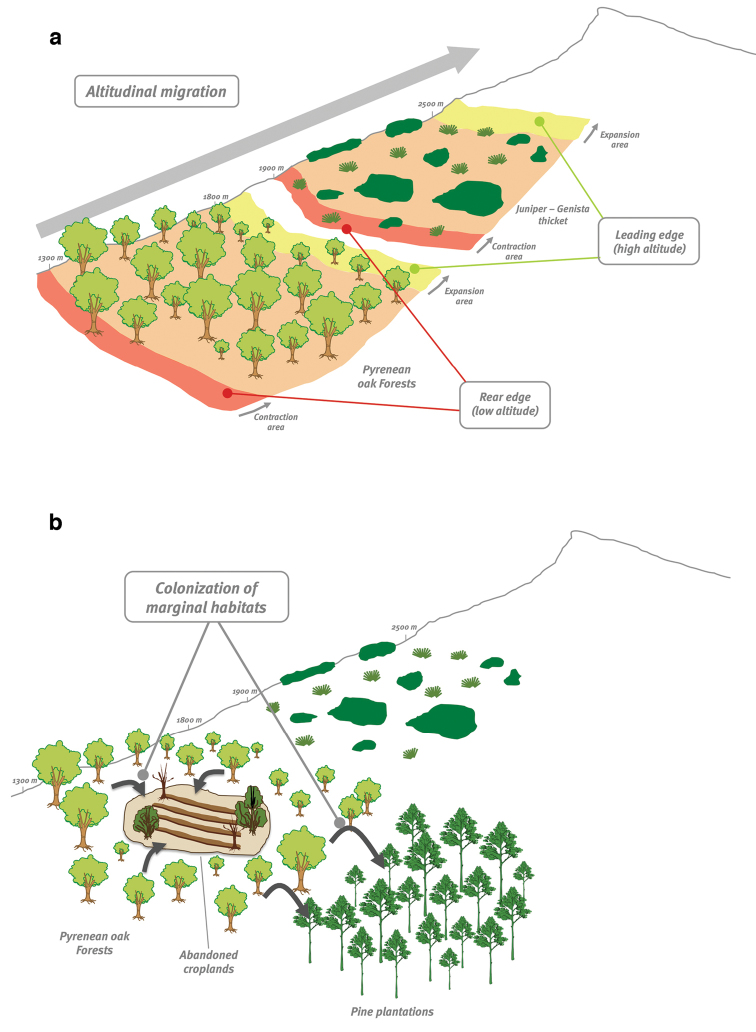
Schematic representation of the two main hypothesis of the project: altitudinal migration (**a**) and colonization of marginal areas (**b**) of *Quercus pyrenaica* forests.

Derived from the two global-change drivers, we have considered two main hypothesis (Figure 1):

***Altitudinal migration*** hypothesis

Several studies have pointed out a trend towards higher temperatures and lower precipitation for the Mediterranean area (Giorgi and Lionello 2008, García-Ruiz et al. 2011). Climate projections forecast an increase of +4.8 °C at the end of the 21st century (Benito et al. 2011) for Sierra Nevada. In this context, shifts in the altitudinal (and latitudinal) distribution of species and communities are expected (Thuiller et al. 2008, Gottfried et al. 2012).

We hypothesised that the range shift of *Quercus pyrenaica* in Sierra Nevada is changing as a consequence of recent changes to temperature, and we would expect an upward expansion (Figure 1a).

***Marginal habitat colonization*** hypothesis

In Mediterranean area, cropland abandonment has been widespread during the second half of the last century (Valbuena-Carabaña et al. 2010, Pías et al. 2014). Land-use change models predict an increase in this trend in the future (Rounsevell et al. 2006). In fact, land abandonment is considered one of the most powerful global-change drivers in developed countries (Escribano-Avila et al. 2012).

We hypothesised that the land-use changes in high mountain (e.g. abandonment of croplands, management of pine plantations) should facilitate the native forest regeneration, and a process of colonization of marginal habitat (abandoned cropland, pine plantations) will occur (Figure 1b).

Overall, focusing on changes will occur in altitudinal migration and/or colonization of marginal habitats, we examine the following questions: Are altitudinal changes in Pyrenean oak forests associated with recent climate changes? Are they more consistent with changes in land use, or are they consistent with both global-change drivers?

### Data published through GBIF

http://www.gbif.es/ipt/resource.do?r=migrame

## Taxonomic coverage

This dataset includes records of the phylum Magnoliophyta (3823 records, 99.58%) and marginally Pinophyta (16 records, below 1% of total records). Most of the records included in this dataset belong to the class Magnoliopsida (99.58%). There are 5 orders represented in the dataset, with Fagales (98.98%) being the most important order. The other 4 orders (Rosales, Cupressales, Sapindales and Pinales) represent only 1.02% of the records. In this collection, 5 families are represented: Fagaceae, Rosaceae, Cupressaceae, Pinaceae, and Sapindaceae. The most represented taxa are *Quercus pyrenaica* Willd. and *Quercus ilex* L. (81.74 and 17.24%, respectively). Of the six taxa included on the dataset, three are considered threatened (Table 1).

**Table 1.** Conservation status and threats of the species included in the dataset.

Scientific Name	Andalusian Red List^1^	IUCN^2^	Threat^3^
Acer opalus subsp. granatense (Boiss.) Font Quer & Rothm.	NT	VU	1,2,3
*Quercus pyrenaica* Willd.	NT	LR-cd	1,2,4,5,6
*Sorbus aria* Wimm.	NT	VU	1,2,3,7

^1^ 2005 Red List of vascular flora of Andalusia (Cabezudo et al. 2005). ^2^ IUCN category in Sierra Nevada (Blanca et al. 1998, Blanca et al. 2001, IUCN 2001, Lorite et al. 2007). ^3^ Threats against the species (Herrera et al. 2000, Prados et al. 2000, Vivero et al. 2000, Marañón et al. 2004, Cabezudo et al. 2005, Gómez-Aparicio et al. 2005, Gómez-Aparicio et al. 2008). *1*: regeneration; *2*: fire; *3*: overgrazing; *4*: inappropriate forestry practices; *5*: changes in agriculture and agricultural practices; *6*: erosion; *7*: demography. VU : Vulnerable ; NT : Near threatened ; LR-nt: LR-cd : Lower Risk-Conservation Dependent .

## Taxonomic ranks

**Kingdom**: Plantae

**Phylum**: Magnoliophyta, Pinophyta

**Class**: Magnoliopsida (Dicotyledones), Pinopsida

**Order**: Fagales, Pinales, Cupressales, Sapindales, Rosales

**Family**: Fagaceae, Pinaceae, Cupressaceae, Sapindaceae, Rosaceae

**Genus**: *Quercus*, *Pinus*, *Juniperus*, *Acer*, *Sorbus*

**Species**: *Quercus pyrenaica*, *Pinus sylvestris*, *Juniperus communis*, Acer opalus subsp. granatense, *Sorbus aria*, *Quercus ilex*

## Spatial coverage

### General spatial coverage

**Quercus pyrenaica forests**

**Figure 2.**
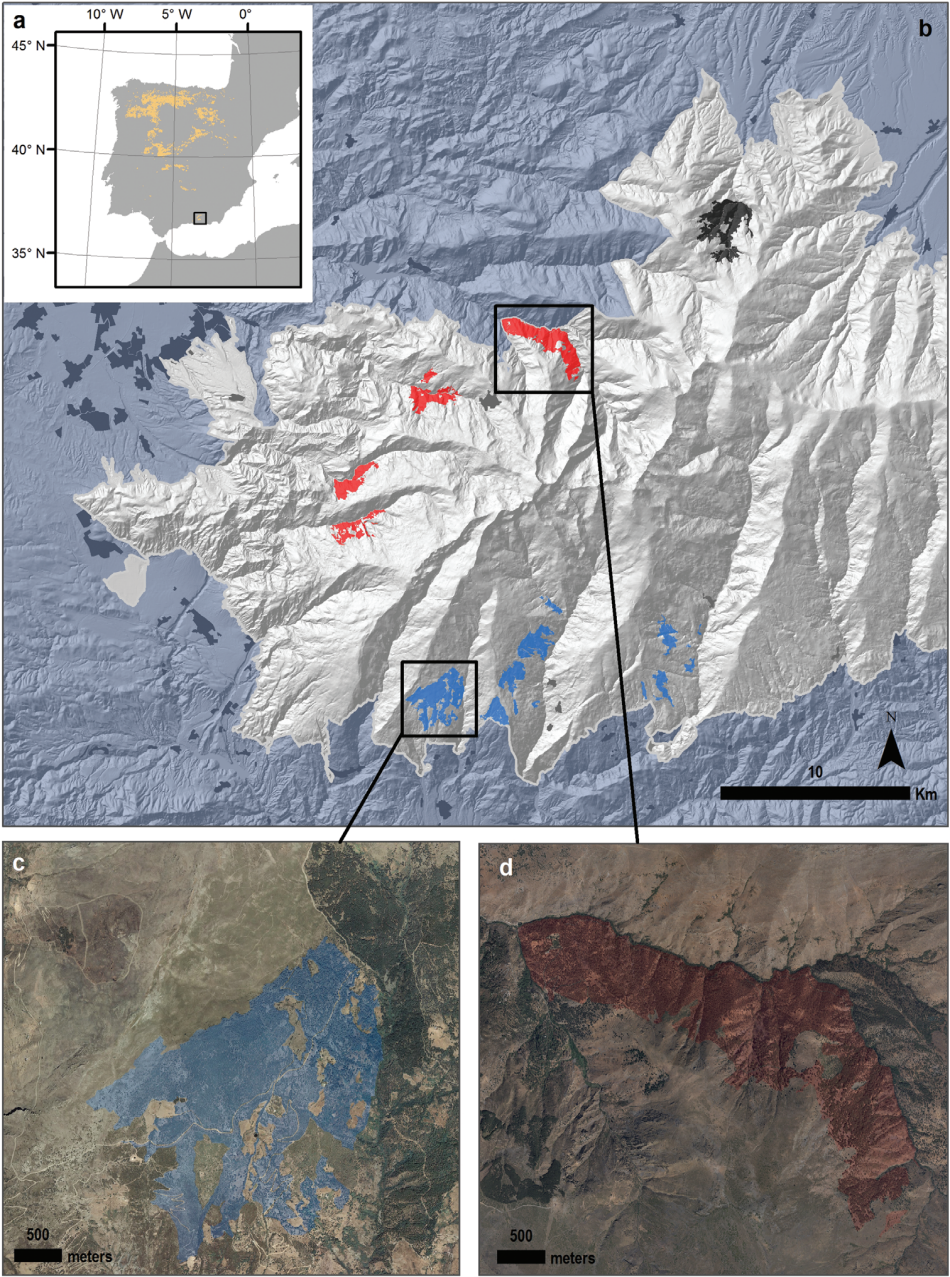
Distribution of *Quercus pyrenaica* forests in Iberian Peninsula (**a**). Sierra Nevada harbours eight populations of *Quercus pyrenaica* clustered into three groups (different colours). We selected two study sites: Robledal de Cañar (**c**) and Robledal San Juan (**d**). Colour Orthophotography of 2009 from Regional Ministry of the Environment, Regional Government of Andalusia.

The Pyrenean oak (*Quercus pyrenaica* Willd.) forests extend through south-western France and the Iberian Peninsula (Franco 1990) (Figure 2a) reaching its southern limit in north of Morocco. In the Iberian Peninsula these forests live under meso-supramediterranean and mesotemperate areas and subhumid, humid and hyperhumid ombroclimate (Rivas-Martínez et al. 2002) living on siliceous soils, or soils poor in basic ions (Vilches de la Serna 2014). *Quercus pyrenaica* requires between 650 and 1200 mm of annual precipitation and a summer minimal precipitation between 100 and 200 mm (Martínez-Parras and Molero-Mesa 1982, García and Jiménez 2009), summer rainfall being a key factor in the distribution of the species (Gavilán et al. 2007, Río et al. 2007).

The forests dominated by *Quercus pyrenaica* constitute an ecosystem included in the Annex I of the Habitat Directive (habitat code 9230: *Quercus pyrenaica oak woods and Quercus robur and Quercus pyrenaica oak woods from Iberian northwestern*). The conservation status of this habitat is not well known (EIONET 2014), partly due to lack of detailed ecological studies (García and Jiménez 2009).

This species reaches its southernmost European limit at Sierra Nevada mountains, where eight oak patches (2400 Has) have been identified (Figure 2b), ranging between 1100 and 2000 m a.s.l. and generally associated to major river valleys. Sierra Nevada is considered a glacial refugia for deciduous *Quercus* species during glaciation (Brewer et al. 2002, Olalde et al. 2002, Rodríguez-Sánchez et al. 2010) and these populations are considered as a rear edge of the habitat distribution, which is important in determining habitat responses to expected climate change (Hampe and Petit 2005).

These forests, like other vegetation types, have undergone intense human pressure (wood cutting, grazing, etc.) which has reduced their distribution area and in some cases has altered their floristic pattern (Gavilán et al. 2000, Gavilán et al. 2007).

*Quercus pyrenaica* is considered as vulnerable in southern Spain (Vivero et al. 2000). The populations of Pyrenean oak forests at Sierra Nevada are considered relict forests (Melendo and Valle 2000, Vivero et al. 2000), undergoing intensive anthropic use in the last few decades (Camacho-Olmedo et al. 2002, Valbuena-Carabaña et al. 2010). The relict presence of this species in Sierra Nevada is related both to its genetic resilience as well as to its high intraspecific genetic diversity (Valbuena-Carabaña and Gil 2013). However, they are also expected to suffer the impact of climate change, due to their climate requirements (wet summers). Thus, simulations of the climate change effects on this habitat forecast a reduction in suitable habitats for Sierra Nevada (Benito et al. 2011).

### Coordinates

36°56'13.2"N and 37°8'9.6"N Latitude; 3°26'16.8"W and 3°20'16.8"W Longitude

### Temporal coverage

2012–2014

### Collection name

Dataset of MIGRAME Project (Global Change, Altitudinal Range Shift and Colonization of Degraded Habitats in Mediterranean Mountains)

### Collection identifier

http://www.gbif.es/ipt/resource.do?r=migrame

## Methods

### Study extent description

The MIGRAME dataset covers the Pyrenean oak forests (see *Spatial coverage* section) in Sierra Nevada mountain range (see *Study area descriptions* section).

### Sampling description

We sampled two localities of the Pyrenean oak forests in Sierra Nevada: Robledal de Cañar and Robledal de San Juan. We selected those two sites based on previous works (Pérez-Luque 2011, Pérez-Luque et al. 2013) that clustered the populations of *Quercus pyrenaica* forests based on their plant species composition and environmental features. The Robledal de Cañar site (Figure 2c) (1366-1935 m a.s.l., 37°57'28.04"N, 3°25'57.1"W; Cáñar, Granada, SE Spain) was located in the Alpujarras Region on the southern slopes of Sierra Nevada. The Robledal de San Juan (Figure 2d) (1189-1899 m a.s.l., 37°7'29.63"N, 3°21'54.60"W; Güejar-Sierra, Granada, SE Spain) site was located in the northern slopes of Sierra Nevada.

The sampling design was determined by the hypothesis of the project (see *Project Design description* section).

**Altitudinal migration design**

**Figure 3.**
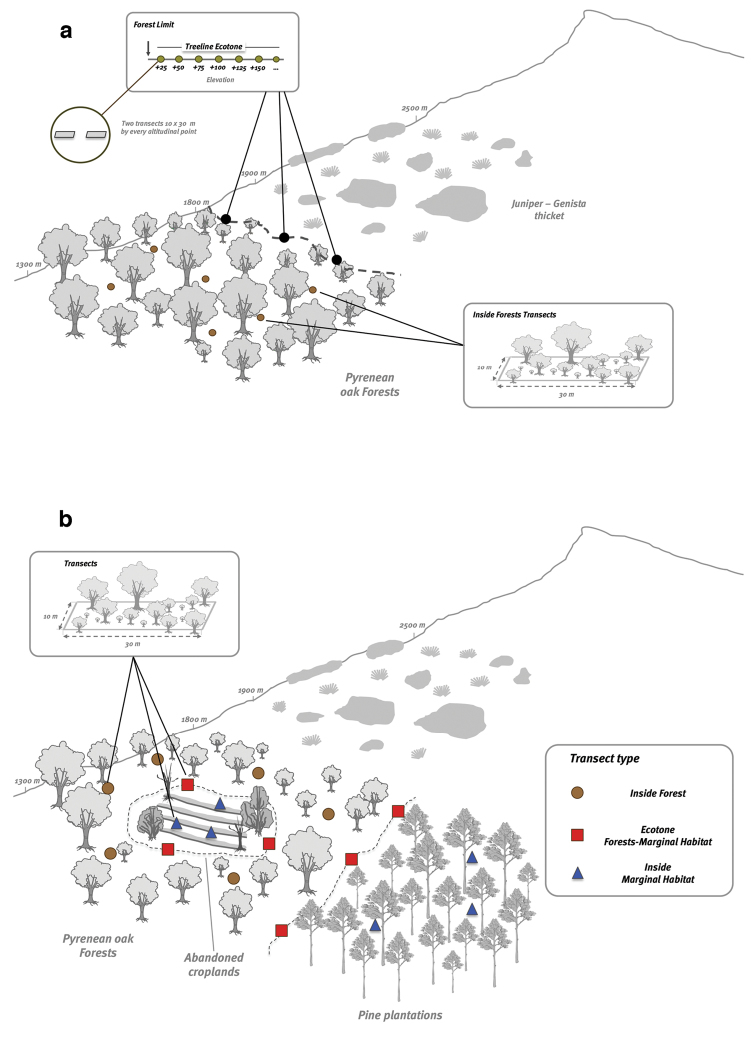
Sampling Design. **a** Altitudinal migration hypothesis. At each study site, from the forest edge to treeline ecotone, we sampled each 25 m of elevation **b** Colonization of marginal habitat hypothesis. Transects were located on three habitat types: Forests (brown circles), Forest Edges (red squares) and Inside Marginal Habitats (blue triangles).

To test our hypothesis of altitudinal migration, we sampled a total of 104 transects (Table 2) distributed along an altitudinal gradient at the two sites. We sampled two transects (at least 10 m apart) every 25 m of elevation from forest limit to treeline ecotone at both study sites. At each locality, we performed three replicates of this design (Figure 3a).

**Table 2.** Transect number of the *Altitudinal migration* design.

Locality	Altitudinal gradient	Transects^1^		
		*R1*	*R2*	*R3*
Robledal de Cañar	1900–2150	12	20	20
Robledal de San Juan	1775–2000	18	18	16

1 For each replicate (R1 to R3) the number of transects is shown.

**Habitat colonization design**

To test the hypothesis of *colonization of marginal habitats*, we laid out transects in two types of marginal habitats: *abandoned agricultural areas* and *pine plantations* (Figure 3b). A total of 64 transects were located within the marginal habitat and on the edge between marginal habitat and Pyrenean oak forest. The number of transects inside the marginal habitat was determined by the size of the marginal habitat (Table 3).

**Table 3.** Transects number of the *Colonization of marginal habitat* design.

				Transects	
Locality	Marginal habitat	Replicate	Surface (ha)	Inside	Edge
Robledal de Cañar	Abandoned Cropland	R1	3.29	6	3
		R2	5.80	9	3
		R3	1.55	3	3
	Pine plantation		80.70	6	6
Robledal de San Juan	Abandoned Cropland	R1	3.46	6	3
		R2	10.36	13	3

**Forest samplings**

In addition to the above surveys, we conducted a survey inside *Quercus pyrenaica* forests. A total of 31 transects were distributed at the two sites.

## Data collection

**Figure 4.**
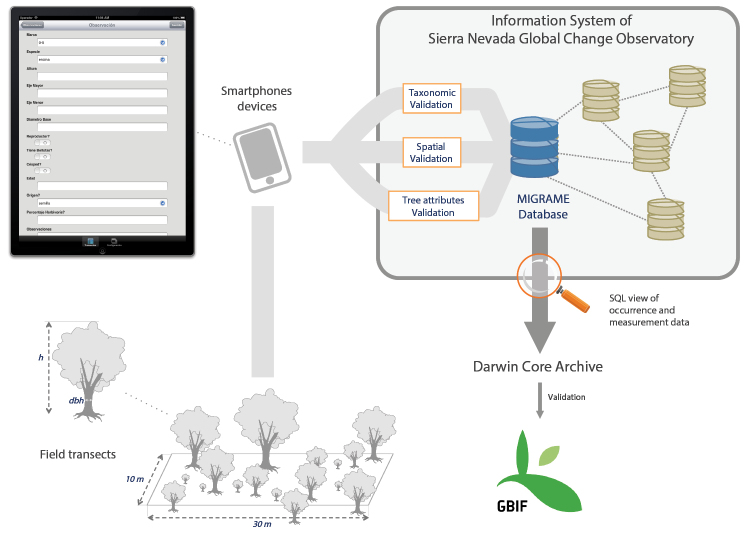
Diagram of integration of the dataset within Information System of Sierra Nevada Global Change Observatory (http://obsnev.es/linaria.html). Field data were recorded with Smartphone devices (see Pérez-Pérez et al. 2013). After a validation process (see Quality Control section) the occurrence and measurement data were accommodated to Darwin Core Archive and integrated into GBIF.

We sampled a total of 199 linear transects of 30 m × 10 m (Suppl. material 1). Within each transect, all tree species were recorded and the species identity was recorded. Diameter size and tree height were measured for all individuals. Field data were recorded using handheld PDAs. A customized application (app) (Figure 4) was built to facilitate both data collection and storage (Pérez-Pérez et al. 2013 – http://obsnev.es/noticia.html?id=4513). The data were automatically integrated into an information system using this application.

### Method step description

All data were stored in a relational database (PostgreSQL) and added to the Information System of Sierra Nevada Global-Change Observatory (Figure 4) (http://obsnev.es/linaria.html – Pérez-Pérez et al. 2012; Free access upon registration). Taxonomic and spatial validations were made on this database (see *Quality-control description*). A custom-made SQL view of the database was performed to gather occurrence data and other variables associated with some occurrence data (diameter size and tree height of each individual).

The occurrence and measurement data were accommodated to fulfil the Darwin Core Standard (Wieczorek et al. 2009, Wieczorek et al. 2012). We used Darwin Core Archive Validator tool (http://tools.gbif.org/dwca-validator/) to check whether the dataset met Darwin Core specifications. The Integrated Publishing Toolkit (IPT v2.0.5) (Robertson et al. 2014) of the Spanish node of the Global Biodiversity Information Facility (GBIF) (http://www.gbif.es/ipt) was used both to upload the Darwin Core Archive and to fill out the metadata.

The Darwin Core elements for the occurrence data included in the dataset were: occurrenceId, modified, language, institutionCode, collectionCode, basisOfRecord, catalogNumber, recordedBy, eventDate, day, month, year, continent, country, countryCode, stateProvince, county, locality, minimumElevationInMeters, maximumElevationInMeters, decimalLongitude, decimalLatitude, coordinateUncertaintyinMeters, geodeticDatum, scientificName, kingdom, phylum, class, order, family, genus, specificEpithet, infraspecificEpithet, scientificNameAuthorship.

For the measurement data, the Darwin Core elements included were: occurrenceId, measurementID, measurementType, measurementValue, measurementAccuracy, measurementUnit, measurementDeterminedDate, measurementDeterminedBy, measurementMethod.

### Quality control description

Transects coordinates were recorded with a handheld Garmin eTrex Vista Global Positioning System (GPS, ±5 m accuracy, Garmin (2007)) (WGS84 Datum). We also used colour digital orthophotographs provided by the Andalusian Cartography Institute and GIS (ArcGIS 9.2; ESRI, Redlands, California, USA) to verify the geographical coordinates of each sampling plot (Chapman and Wieczorek 2006).

The specimens were taxonomically identified using Flora iberica (Castroviejo 1986–2005). The scientific names were checked with databases of International Plant Names Index (IPNI 2013) and Catalogue of Life/Species 2000 (Roskov et al. 2015). We also used the R package taxize (Chamberlain and Szöcs 2013, Chamberlain et al. 2014) to verify the taxonomical classification.

We also performed validation procedures (Chapman 2005a, 2005b) (geographic coordinate format, coordinates within country/provincial boundaries, absence of ASCII anomalous characters in the dataset) with DARWIN_TEST (v3.2) software (Ortega-Maqueda and Pando 2008).

## Dataset description

**Object name**: Darwin Core Archive Dataset of MIGRAME Project (Global Change, Altitudinal Range Shift and Colonization of Degraded Habitats in Mediterranean Mountains)

**Character encoding**: UTF-8

**Format name**: Darwin Core Archive format

**Format version**: 1.0

Distribution: http://www.gbif.es/ipt/resource.do?r=migrame

**Publication date of data**: 2015-05-13

**Language**: English

**Licenses of use**: This “Dataset of MIGRAME Project (Global Change, Altitudinal Range Shift and Colonization of Degraded Habitats in Mediterranean Mountains)” is licensed under a made available under the Creative Commons Attribution Non Commercial (CC-BY-NC) 4.0 License http://creativecommons.org/licenses/by-nc/4.0/legalcode

**Metadata language**: English

**Date of metadata creation**: 2015-05-13

**Hierarchy level**: DataSet

## Supplementary materials

Supplementary material 1 Table S1 Data type: Table Explanation note: Information about transects of the project. Elevation in m a.s.l. Type: AM = Altitudinal migration ; FO = Forest ; MH = Marginal Habitat . Subtype: AC-e : Abandoned Cropland: edge ; AC-i : Abandoned Cropland: inside ; Pp-e : Pine plantations: edge ; Pp-i : Pine plantations: inside ; TE : Treeline Ecotone . Locality: CA = Robledal de Cáñar ; SJ = Robledal de San Juan . This dataset is made available under the Open Database License (http://opendatacommons.org/licenses/odbl/1.0/). The Open Database License (ODbL) is a license agreement intended to allow users to freely share, modify, and use this Dataset while maintaining this same freedom for others, provided that the original source and author(s) are credited.Antonio Jesús Pérez-Luque, Regino Zamora, Francisco Javier Bonet, Ramón Pérez-Pérez
