# Supplementary material for: Dataset of MIGRAME Project (Global Change, Altitudinal Range Shift and Colonization of Degraded Habitats in Mediterranean Mountains)
Source: PhytoKeys. 2015 Oct 1;(56):61–81. doi: 10.3897/phytokeys.56.5482 (PMC4611749; doi:10.3897/phytokeys.56.5482)
Supplement: Supplementary material 1 — Table S1 [file phytokeys-056-061-s001.docx]

Supplementary Material.

Table S1. Information about transects of the project. Elevation in m *a.s.l.*. Type: *AM* = Altitudinal migration; *FO* = Forest; *MH* = Marginal Habitat. Subtype: *AC-e:* Abandoned Cropland: edge; *AC-i* = Abandoned Cropland: inside; *Pp-e:* Pine plantations: edge; *Pp-i:* Pine plantations: inside; *TE* = Treeline Ecotone. Locality: *CA* = Robledal de Cáñar; *SJ* = Robledal de San Juan.

| Transect name | Longitude | Latitude | Elevation | Type | Subtype | Locality | County | Province | Country |
| --- | --- | --- | --- | --- | --- | --- | --- | --- | --- |
| G032b | -3.353 | 37.12 | 1848 | AM | TE | SJ | Güejar-Sierra | Granada | Spain |
| A009 | -3.356 | 37.12 | 1718 | MH | AC-i | SJ | Güejar-Sierra | Granada | Spain |
| G038a | -3.355 | 37.11 | 2001 | AM | TE | SJ | Güejar-Sierra | Granada | Spain |
| G036a | -3.354 | 37.11 | 1947 | AM | TE | SJ | Güejar-Sierra | Granada | Spain |
| G035a | -3.353 | 37.11 | 1924 | AM | TE | SJ | Güejar-Sierra | Granada | Spain |
| G013b | -3.361 | 37.12 | 1970 | AM | TE | SJ | Güejar-Sierra | Granada | Spain |
| G006a | -3.360 | 37.12 | 1804 | AM | TE | SJ | Güejar-Sierra | Granada | Spain |
| G024a | -3.355 | 37.12 | 1915 | AM | TE | SJ | Güejar-Sierra | Granada | Spain |
| G007a | -3.360 | 37.12 | 1828 | AM | TE | SJ | Güejar-Sierra | Granada | Spain |
| G012b | -3.361 | 37.12 | 1943 | AM | TE | SJ | Güejar-Sierra | Granada | Spain |
| G008a | -3.360 | 37.12 | 1850 | AM | TE | SJ | Güejar-Sierra | Granada | Spain |
| G023a | -3.355 | 37.12 | 1897 | AM | TE | SJ | Güejar-Sierra | Granada | Spain |
| G009a | -3.360 | 37.12 | 1876 | AM | TE | SJ | Güejar-Sierra | Granada | Spain |
| G037a | -3.354 | 37.11 | 1973 | AM | TE | SJ | Güejar-Sierra | Granada | Spain |
| G010a | -3.360 | 37.12 | 1901 | AM | TE | SJ | Güejar-Sierra | Granada | Spain |
| G009b | -3.360 | 37.12 | 1876 | AM | TE | SJ | Güejar-Sierra | Granada | Spain |
| B006 | -3.435 | 36.96 | 1835 | MH | AC-e | CA | Cáñar | Granada | Spain |
| T050 | -3.424 | 36.97 | 2101 | AM | TE | CA | Cáñar | Granada | Spain |
| T053 | -3.428 | 36.97 | 2108 | AM | TE | CA | Cáñar | Granada | Spain |
| T054 | -3.425 | 36.97 | 2144 | AM | TE | CA | Cáñar | Granada | Spain |
| T057 | -3.429 | 36.97 | 2141 | AM | TE | CA | Cáñar | Granada | Spain |
| T018b | -3.428 | 36.96 | 1921 | AM | TE | CA | Cáñar | Granada | Spain |
| T022b | -3.431 | 36.97 | 2021 | AM | TE | CA | Cáñar | Granada | Spain |
| T017a | -3.426 | 36.96 | 1899 | AM | TE | CA | Cáñar | Granada | Spain |
| T014a | -3.422 | 36.97 | 2033 | AM | TE | CA | Cáñar | Granada | Spain |
| T021b | -3.430 | 36.97 | 1998 | AM | TE | CA | Cáñar | Granada | Spain |
| T019b | -3.429 | 36.97 | 1947 | AM | TE | CA | Cáñar | Granada | Spain |
| T020b | -3.430 | 36.97 | 1972 | AM | TE | CA | Cáñar | Granada | Spain |
| G011a | -3.360 | 37.12 | 1920 | AM | TE | SJ | Güejar-Sierra | Granada | Spain |
| B001 | -3.418 | 36.97 | 1889 | MH | AC-e | CA | Cáñar | Granada | Spain |
| G037b | -3.355 | 37.11 | 1978 | AM | TE | SJ | Güejar-Sierra | Granada | Spain |
| B003 | -3.418 | 36.97 | 1874 | MH | AC-e | CA | Cáñar | Granada | Spain |
| G036b | -3.354 | 37.11 | 1944 | AM | TE | SJ | Güejar-Sierra | Granada | Spain |
| G038b | -3.355 | 37.11 | 2003 | AM | TE | SJ | Güejar-Sierra | Granada | Spain |
| T013b | -3.421 | 36.97 | 2009 | AM | TE | CA | Cáñar | Granada | Spain |
| T013a | -3.422 | 36.97 | 2007 | AM | TE | CA | Cáñar | Granada | Spain |
| T011a | -3.421 | 36.97 | 1955 | AM | TE | CA | Cáñar | Granada | Spain |
| T011b | -3.421 | 36.97 | 1956 | AM | TE | CA | Cáñar | Granada | Spain |
| T012b | -3.421 | 36.97 | 1981 | AM | TE | CA | Cáñar | Granada | Spain |
| T010b | -3.420 | 36.97 | 1935 | AM | TE | CA | Cáñar | Granada | Spain |
| T007b | -3.426 | 36.97 | 2054 | AM | TE | CA | Cáñar | Granada | Spain |
| T007a | -3.426 | 36.97 | 2054 | AM | TE | CA | Cáñar | Granada | Spain |
| T021a | -3.431 | 36.97 | 1996 | AM | TE | CA | Cáñar | Granada | Spain |
| T006a | -3.425 | 36.97 | 2030 | AM | TE | CA | Cáñar | Granada | Spain |
| P004 | -3.421 | 36.96 | 1838 | MH | AC-i | CA | Cáñar | Granada | Spain |
| T010a | -3.420 | 36.97 | 1934 | AM | TE | CA | Cáñar | Granada | Spain |
| T008b | -3.426 | 36.97 | 2080 | AM | TE | CA | Cáñar | Granada | Spain |
| C002 | -3.365 | 37.12 | 1673 | MH | AC-e | SJ | Güejar-Sierra | Granada | Spain |
| C006 | -3.356 | 37.13 | 1641 | MH | AC-e | SJ | Güejar-Sierra | Granada | Spain |
| A018 | -3.355 | 37.12 | 1720 | MH | AC-i | SJ | Güejar-Sierra | Granada | Spain |
| P012 | -3.423 | 36.96 | 1853 | MH | AC-i | CA | Cáñar | Granada | Spain |
| A015 | -3.354 | 37.12 | 1689 | MH | AC-i | SJ | Güejar-Sierra | Granada | Spain |
| A013 | -3.356 | 37.12 | 1652 | MH | AC-i | SJ | Güejar-Sierra | Granada | Spain |
| P028 | -3.433 | 36.96 | 1845 | FO | F | CA | Cáñar | Granada | Spain |
| P017 | -3.420 | 36.96 | 1823 | MH | AC-i | CA | Cáñar | Granada | Spain |
| P010 | -3.433 | 36.96 | 1798 | MH | AC-i | CA | Cáñar | Granada | Spain |
| P009 | -3.434 | 36.96 | 1809 | MH | AC-i | CA | Cáñar | Granada | Spain |
| P031 | -3.430 | 36.96 | 1815 | FO | F | CA | Cáñar | Granada | Spain |
| B005 | -3.433 | 36.96 | 1800 | MH | AC-e | CA | Cáñar | Granada | Spain |
| B011 | -3.420 | 36.96 | 1842 | MH | AC-e | CA | Cáñar | Granada | Spain |
| A010 | -3.356 | 37.12 | 1706 | MH | AC-i | SJ | Güejar-Sierra | Granada | Spain |
| A004 | -3.364 | 37.13 | 1588 | MH | AC-i | SJ | Güejar-Sierra | Granada | Spain |
| A006 | -3.364 | 37.13 | 1612 | MH | AC-i | SJ | Güejar-Sierra | Granada | Spain |
| A008 | -3.356 | 37.12 | 1684 | MH | AC-i | SJ | Güejar-Sierra | Granada | Spain |
| P002 | -3.434 | 36.96 | 1805 | MH | AC-i | CA | Cáñar | Granada | Spain |
| C010 | -3.355 | 37.13 | 1649 | MH | AC-e | SJ | Güejar-Sierra | Granada | Spain |
| A029 | -3.357 | 37.13 | 1575 | FO | F | SJ | Güejar-Sierra | Granada | Spain |
| A026 | -3.358 | 37.12 | 1641 | FO | F | SJ | Güejar-Sierra | Granada | Spain |
| T008a | -3.427 | 36.97 | 2079 | AM | TE | CA | Cáñar | Granada | Spain |
| D015 | -3.411 | 36.97 | 1861 | MH | Pp-i | CA | Cáñar | Granada | Spain |
| T022a | -3.431 | 36.97 | 2022 | AM | TE | CA | Cáñar | Granada | Spain |
| D019 | -3.410 | 36.97 | 1751 | MH | Pp-i | CA | Cáñar | Granada | Spain |
| D018 | -3.409 | 36.97 | 1747 | MH | Pp-i | CA | Cáñar | Granada | Spain |
| D003p | -3.413 | 36.97 | 1805 | MH | Pp-e | CA | Cáñar | Granada | Spain |
| T016b | -3.423 | 36.97 | 2084 | AM | TE | CA | Cáñar | Granada | Spain |
| T016a | -3.424 | 36.97 | 2082 | AM | TE | CA | Cáñar | Granada | Spain |
| T003a | -3.424 | 36.97 | 1947 | AM | TE | CA | Cáñar | Granada | Spain |
| T004a | -3.425 | 36.97 | 1978 | AM | TE | CA | Cáñar | Granada | Spain |
| T017b | -3.426 | 36.96 | 1898 | AM | TE | CA | Cáñar | Granada | Spain |
| T020a | -3.430 | 36.97 | 1972 | AM | TE | CA | Cáñar | Granada | Spain |
| T005a | -3.425 | 36.97 | 2003 | AM | TE | CA | Cáñar | Granada | Spain |
| P008 | -3.433 | 36.96 | 1807 | MH | AC-i | CA | Cáñar | Granada | Spain |
| P037 | -3.422 | 36.96 | 1806 | FO | F | CA | Cáñar | Granada | Spain |
| P014 | -3.422 | 36.96 | 1845 | MH | AC-i | CA | Cáñar | Granada | Spain |
| D002r | -3.413 | 36.97 | 1777 | MH | Pp-e | CA | Cáñar | Granada | Spain |
| G021a | -3.354 | 37.12 | 1847 | AM | TE | SJ | Güejar-Sierra | Granada | Spain |
| G014b | -3.353 | 37.12 | 1799 | AM | TE | SJ | Güejar-Sierra | Granada | Spain |
| G022a | -3.354 | 37.12 | 1872 | AM | TE | SJ | Güejar-Sierra | Granada | Spain |
| G020b | -3.353 | 37.12 | 1813 | AM | TE | SJ | Güejar-Sierra | Granada | Spain |
| T001a | -3.423 | 36.97 | 1904 | AM | TE | CA | Cáñar | Granada | Spain |
| T005b | -3.425 | 36.97 | 2005 | AM | TE | CA | Cáñar | Granada | Spain |
| T018a | -3.428 | 36.96 | 1919 | AM | TE | CA | Cáñar | Granada | Spain |
| T015b | -3.423 | 36.97 | 2059 | AM | TE | CA | Cáñar | Granada | Spain |
| T002b | -3.423 | 36.97 | 1919 | AM | TE | CA | Cáñar | Granada | Spain |
| T002a | -3.424 | 36.97 | 1918 | AM | TE | CA | Cáñar | Granada | Spain |
| T009b | -3.419 | 36.97 | 1912 | AM | TE | CA | Cáñar | Granada | Spain |
| T003b | -3.424 | 36.97 | 1949 | AM | TE | CA | Cáñar | Granada | Spain |
| T015a | -3.423 | 36.97 | 2057 | AM | TE | CA | Cáñar | Granada | Spain |
| T012a | -3.421 | 36.97 | 1979 | AM | TE | CA | Cáñar | Granada | Spain |
| T004b | -3.424 | 36.97 | 1980 | AM | TE | CA | Cáñar | Granada | Spain |
| T014b | -3.422 | 36.97 | 2035 | AM | TE | CA | Cáñar | Granada | Spain |
| T001b | -3.423 | 36.97 | 1904 | AM | TE | CA | Cáñar | Granada | Spain |
| T009a | -3.419 | 36.97 | 1913 | AM | TE | CA | Cáñar | Granada | Spain |
| T006b | -3.425 | 36.97 | 2031 | AM | TE | CA | Cáñar | Granada | Spain |
| T019a | -3.430 | 36.96 | 1950 | AM | TE | CA | Cáñar | Granada | Spain |
| P007 | -3.423 | 36.96 | 1793 | FO | F | CA | Cáñar | Granada | Spain |
| P003 | -3.434 | 36.96 | 1830 | MH | AC-i | CA | Cáñar | Granada | Spain |
| P001 | -3.434 | 36.96 | 1811 | MH | AC-i | CA | Cáñar | Granada | Spain |
| P027 | -3.422 | 36.96 | 1849 | FO | F | CA | Cáñar | Granada | Spain |
| D002p | -3.413 | 36.97 | 1778 | MH | Pp-e | CA | Cáñar | Granada | Spain |
| P005 | -3.418 | 36.97 | 1878 | MH | AC-i | CA | Cáñar | Granada | Spain |
| P006 | -3.418 | 36.97 | 1874 | MH | AC-i | CA | Cáñar | Granada | Spain |
| P020 | -3.418 | 36.97 | 1889 | MH | AC-i | CA | Cáñar | Granada | Spain |
| P024 | -3.420 | 36.97 | 1858 | FO | F | CA | Cáñar | Granada | Spain |
| P034 | -3.417 | 36.97 | 1851 | FO | F | CA | Cáñar | Granada | Spain |
| P013 | -3.421 | 36.96 | 1804 | MH | AC-i | CA | Cáñar | Granada | Spain |
| P016 | -3.421 | 36.96 | 1824 | MH | AC-i | CA | Cáñar | Granada | Spain |
| P015 | -3.421 | 36.96 | 1832 | MH | AC-i | CA | Cáñar | Granada | Spain |
| P018 | -3.421 | 36.96 | 1840 | MH | AC-i | CA | Cáñar | Granada | Spain |
| P011 | -3.420 | 36.96 | 1817 | MH | AC-i | CA | Cáñar | Granada | Spain |
| P038 | -3.425 | 36.96 | 1789 | FO | F | CA | Cáñar | Granada | Spain |
| P033 | -3.425 | 36.96 | 1843 | FO | F | CA | Cáñar | Granada | Spain |
| P030 | -3.431 | 36.96 | 1866 | FO | F | CA | Cáñar | Granada | Spain |
| P035 | -3.432 | 36.96 | 1796 | FO | F | CA | Cáñar | Granada | Spain |
| D014 | -3.414 | 36.97 | 1883 | MH | Pp-i | CA | Cáñar | Granada | Spain |
| D016 | -3.410 | 36.97 | 1819 | MH | Pp-i | CA | Cáñar | Granada | Spain |
| D017 | -3.410 | 36.97 | 1774 | MH | Pp-i | CA | Cáñar | Granada | Spain |
| D003r | -3.414 | 36.97 | 1804 | MH | Pp-e | CA | Cáñar | Granada | Spain |
| D001p | -3.411 | 36.97 | 1736 | MH | Pp-e | CA | Cáñar | Granada | Spain |
| D001r | -3.412 | 36.97 | 1736 | MH | Pp-e | CA | Cáñar | Granada | Spain |
| P023 | -3.417 | 36.97 | 1868 | FO | F | CA | Cáñar | Granada | Spain |
| P026 | -3.417 | 36.97 | 1892 | FO | F | CA | Cáñar | Granada | Spain |
| P021 | -3.420 | 36.97 | 1883 | FO | F | CA | Cáñar | Granada | Spain |
| P022 | -3.418 | 36.97 | 1833 | FO | F | CA | Cáñar | Granada | Spain |
| B004 | -3.434 | 36.96 | 1802 | MH | AC-e | CA | Cáñar | Granada | Spain |
| B010 | -3.422 | 36.96 | 1851 | MH | AC-e | CA | Cáñar | Granada | Spain |
| B008 | -3.421 | 36.96 | 1847 | MH | AC-e | CA | Cáñar | Granada | Spain |
| B002 | -3.418 | 36.97 | 1873 | MH | AC-e | CA | Cáñar | Granada | Spain |
| G007b | -3.360 | 37.12 | 1830 | AM | TE | SJ | Güejar-Sierra | Granada | Spain |
| G027b | -3.356 | 37.12 | 1973 | AM | TE | SJ | Güejar-Sierra | Granada | Spain |
| G027a | -3.356 | 37.12 | 1977 | AM | TE | SJ | Güejar-Sierra | Granada | Spain |
| G024b | -3.355 | 37.12 | 1916 | AM | TE | SJ | Güejar-Sierra | Granada | Spain |
| G014a | -3.353 | 37.12 | 1802 | AM | TE | SJ | Güejar-Sierra | Granada | Spain |
| G001a | -3.359 | 37.12 | 1775 | AM | TE | SJ | Güejar-Sierra | Granada | Spain |
| G011b | -3.360 | 37.12 | 1920 | AM | TE | SJ | Güejar-Sierra | Granada | Spain |
| G021b | -3.354 | 37.12 | 1845 | AM | TE | SJ | Güejar-Sierra | Granada | Spain |
| G006b | -3.360 | 37.12 | 1799 | AM | TE | SJ | Güejar-Sierra | Granada | Spain |
| G026b | -3.356 | 37.12 | 1950 | AM | TE | SJ | Güejar-Sierra | Granada | Spain |
| G026a | -3.356 | 37.12 | 1954 | AM | TE | SJ | Güejar-Sierra | Granada | Spain |
| G013a | -3.360 | 37.12 | 1970 | AM | TE | SJ | Güejar-Sierra | Granada | Spain |
| G023b | -3.355 | 37.12 | 1891 | AM | TE | SJ | Güejar-Sierra | Granada | Spain |
| G020a | -3.353 | 37.12 | 1822 | AM | TE | SJ | Güejar-Sierra | Granada | Spain |
| G010b | -3.360 | 37.12 | 1901 | AM | TE | SJ | Güejar-Sierra | Granada | Spain |
| G008b | -3.360 | 37.12 | 1852 | AM | TE | SJ | Güejar-Sierra | Granada | Spain |
| G025b | -3.356 | 37.12 | 1932 | AM | TE | SJ | Güejar-Sierra | Granada | Spain |
| G025a | -3.355 | 37.12 | 1936 | AM | TE | SJ | Güejar-Sierra | Granada | Spain |
| G022b | -3.354 | 37.12 | 1865 | AM | TE | SJ | Güejar-Sierra | Granada | Spain |
| G012a | -3.360 | 37.12 | 1941 | AM | TE | SJ | Güejar-Sierra | Granada | Spain |
| G034b | -3.353 | 37.11 | 1896 | AM | TE | SJ | Güejar-Sierra | Granada | Spain |
| G034a | -3.353 | 37.11 | 1896 | AM | TE | SJ | Güejar-Sierra | Granada | Spain |
| G033b | -3.353 | 37.11 | 1874 | AM | TE | SJ | Güejar-Sierra | Granada | Spain |
| G033a | -3.353 | 37.11 | 1880 | AM | TE | SJ | Güejar-Sierra | Granada | Spain |
| G028b | -3.352 | 37.12 | 1812 | AM | TE | SJ | Güejar-Sierra | Granada | Spain |
| G028a | -3.352 | 37.12 | 1820 | AM | TE | SJ | Güejar-Sierra | Granada | Spain |
| G035b | -3.354 | 37.11 | 1925 | AM | TE | SJ | Güejar-Sierra | Granada | Spain |
| G032a | -3.352 | 37.11 | 1853 | AM | TE | SJ | Güejar-Sierra | Granada | Spain |
| C001 | -3.365 | 37.13 | 1621 | MH | AC-e | SJ | Güejar-Sierra | Granada | Spain |
| C003 | -3.364 | 37.13 | 1578 | MH | AC-e | SJ | Güejar-Sierra | Granada | Spain |
| C004 | -3.357 | 37.12 | 1658 | MH | AC-e | SJ | Güejar-Sierra | Granada | Spain |
| A017 | -3.357 | 37.12 | 1681 | MH | AC-i | SJ | Güejar-Sierra | Granada | Spain |
| A019 | -3.354 | 37.13 | 1660 | MH | AC-i | SJ | Güejar-Sierra | Granada | Spain |
| A016 | -3.355 | 37.12 | 1708 | MH | AC-i | SJ | Güejar-Sierra | Granada | Spain |
| A014 | -3.355 | 37.12 | 1672 | MH | AC-i | SJ | Güejar-Sierra | Granada | Spain |
| A012 | -3.356 | 37.12 | 1673 | MH | AC-i | SJ | Güejar-Sierra | Granada | Spain |
| A011 | -3.356 | 37.12 | 1693 | MH | AC-i | SJ | Güejar-Sierra | Granada | Spain |
| A003 | -3.365 | 37.13 | 1607 | MH | AC-i | SJ | Güejar-Sierra | Granada | Spain |
| A002 | -3.364 | 37.13 | 1612 | MH | AC-i | SJ | Güejar-Sierra | Granada | Spain |
| A001 | -3.365 | 37.12 | 1640 | MH | AC-i | SJ | Güejar-Sierra | Granada | Spain |
| A005 | -3.365 | 37.13 | 1649 | MH | AC-i | SJ | Güejar-Sierra | Granada | Spain |
| A007 | -3.354 | 37.12 | 1709 | MH | AC-i | SJ | Güejar-Sierra | Granada | Spain |
| A024 | -3.366 | 37.13 | 1629 | FO | F | SJ | Güejar-Sierra | Granada | Spain |
| A028 | -3.357 | 37.12 | 1604 | FO | F | SJ | Güejar-Sierra | Granada | Spain |
| A027 | -3.358 | 37.12 | 1613 | FO | F | SJ | Güejar-Sierra | Granada | Spain |
| G001b | -3.360 | 37.12 | 1770 | AM | TE | SJ | Güejar-Sierra | Granada | Spain |
| A040 | -3.354 | 37.13 | 1629 | FO | F | SJ | Güejar-Sierra | Granada | Spain |
| A041 | -3.353 | 37.12 | 1746 | FO | F | SJ | Güejar-Sierra | Granada | Spain |
| A036 | -3.357 | 37.12 | 1720 | FO | F | SJ | Güejar-Sierra | Granada | Spain |
| A037 | -3.359 | 37.12 | 1729 | FO | F | SJ | Güejar-Sierra | Granada | Spain |
| A038 | -3.360 | 37.12 | 1652 | FO | F | SJ | Güejar-Sierra | Granada | Spain |
| A039 | -3.360 | 37.12 | 1583 | FO | F | SJ | Güejar-Sierra | Granada | Spain |
| A035 | -3.364 | 37.12 | 1615 | FO | F | SJ | Güejar-Sierra | Granada | Spain |
| A034 | -3.365 | 37.13 | 1507 | FO | F | SJ | Güejar-Sierra | Granada | Spain |
| A033 | -3.365 | 37.13 | 1527 | FO | F | SJ | Güejar-Sierra | Granada | Spain |
| A032 | -3.366 | 37.13 | 1571 | FO | F | SJ | Güejar-Sierra | Granada | Spain |
| A031 | -3.367 | 37.12 | 1674 | FO | F | SJ | Güejar-Sierra | Granada | Spain |
| T051 | -3.424 | 36.97 | 2099 | AM | TE | CA | Cáñar | Granada | Spain |
| T052 | -3.427 | 36.97 | 2105 | AM | TE | CA | Cáñar | Granada | Spain |
| T055 | -3.426 | 36.97 | 2149 | AM | TE | CA | Cáñar | Granada | Spain |
| T056 | -3.428 | 36.97 | 2137 | AM | TE | CA | Cáñar | Granada | Spain |
